# Cohort Profile: the Cuba Prospective Study

**DOI:** 10.1093/ije/dyy297

**Published:** 2019-02-22

**Authors:** Nurys Armas Rojas, Ben Lacey, Sarah Lewington, Patricia Varona Pérez, Julie Ann Burrett, José Manuel Morales Rigau, Paul Sherliker, Jillian Boreham, Osvaldo Jesús Hernández López, Blake Thomson, Fernando Achiong Estupiñan, Mayda Díaz González, Noel Rosquete Muñoz, Marelis Cendra Asencio, Jonathan Emberson, Richard Peto, Alfredo Dueñas Herrera

**Affiliations:** 1 National Institute of Cardiology and Cardiovascular Surgery, Havana, Cuba; 2 Clinical Trial Service Unit and Epidemiological Studies Unit (CTSU), Nuffield Department of Population Health, University of Oxford, Oxford, UK; 3 MRC Population Health Research Unit, University of Oxford, Oxford, UK; 4 Institute of Hygiene, Epidemiology and Microbiology, Cuban Ministry of Public Health, Havana, Cuba; 5 Provincial Centre of Hygiene, Epidemiology and Microbiology, Matanzas, Cuba; 6 Municipal Centre of Hygiene, Epidemiology and Microbiology, Jagüey Grande, Matanzas, Cuba; 7 Municipal Centre of Hygiene, Epidemiology and Microbiology, Colón, Matanzas, Cuba; 8 Municipal Centre of Hygiene, Epidemiology and Microbiology, Camagüey, Camagüey, Cuba

**Profile in a nutshell**

- The Cuba Prospective Study is a cohort study investigating the effects of smoking and other major risk factors on premature adult mortality.
- A total of 146 556 adults aged 30 and over were recruited from the general population in Cuba between 1996 and 2002.
- Participants were interviewed, measured and followed up for cause-specific mortality through electronic linkage to national death registries.
- In 2006–08, 24 345 participants were re-surveyed using the same procedures as at recruitment. These repeat assessments will be used to account for random error and biological variation in measurements taken at baseline.
- After nearly 20 years of follow-up, over 30 000 participants have died, including about 14 000 cardiovascular deaths and 7000 cancer deaths.
- Specific proposals for future collaboration are welcome, addressed to the study’s Cuba-based or Oxford-based investigators.

## Why was the cohort set up?

The Cuba Prospective Study is a cohort study of 146 556 adults aged 30 and over, recruited from the general population in Cuba between 1996 and 2002. The study was established primarily to investigate the effects of tobacco (both cigarettes and cigars) on premature death in Cuba, but includes other major risk factors for chronic disease, such as alcohol consumption, adiposity and blood pressure. These risk factors have been studied extensively in high-income countries, but their effects can vary greatly from one population to another, and there is still substantial uncertainty as to how important these are in different settings and how their importance is changing with time.

In Cuba, there have been substantial reductions over the past few decades in mortality from infectious diseases, such that the chronic diseases of middle age (here defined as age 30–69 years) have become the main causes of premature death.^1^ At 2010 mortality rates (corresponding approximately to the current mid-point of follow-up in this study), one-quarter of Cuban men and one-sixth of Cuban women will die in middle age, predominantly from vascular and neoplastic diseases (Table 1).^2^ This prospective study will provide a clearer understanding of the relevance of smoking and other major risk factors to premature death in Cuba. Importantly, the large size of the study will allow the effects of risk factors to be assessed in population subgroups (e.g. by age and sex) and at different levels of each other (e.g. the combined effect of smoking and drinking).

**Table 1.** Age-standardized mortality rates^a^ in 2010 at ages 30 to 69 for Cuba and, for comparison, USA and UK

	Annual deaths per 100 000					
	Men			Women		
Cause of death	Cuba	USA	UK	Cuba	USA	UK
Vascular	254	206	158	154	99	63
Ischaemic heart disease	151	138	104	81	55	30
Stroke	63	28	23	46	20	16
Other	40	41	31	28	24	17

Neoplastic	241	217	215	191	170	180
Tracheal, bronchus and lung cancer	75	69	56	41	47	40
Upper aero-digestive cancer^b^	29	9	10	5	3	3
Other	137	138	149	145	121	136

Other medical	168	208	136	127	141	94
Chronic obstructive pulmonary disease	22	30	24	22	25	20
Chronic liver disease, mainly cirrhosis	29	37	29	10	17	15
Other	118	141	84	95	99	60
External	81	76	38	23	28	13
						
All causes	744	706	547	496	438	350

^a^Rates are age-standardized by taking the unweighted average of the component 5-year mortality rates (e.g. 30–34, 35–39… 65–69 for the age range 30–69 years). Source: Global Burden of Disease Study 2016.^2^

^b^Includes: lip and oral cavity cancer; larynx cancer; nasopharynx cancer; and other pharynx cancer.

The study was conducted as a collaboration between: the Institute of Cardiology in Havana; the Cuban National Institute of Hygiene, Epidemiology and Microbiology; the Municipal Centers of Hygiene and Epidemiology of Pinar del Río, Matanzas and Camagüey; and the Clinical Trial Service Unit and Epidemiological Studies Unit (CTSU) in Oxford, UK. The study gained approval via the Ethics Committee of the National Institute of Cardiology and Cardiac Surgery, Havana (ref: 0404134).

## Who is in the cohort?

**Figure 1.**
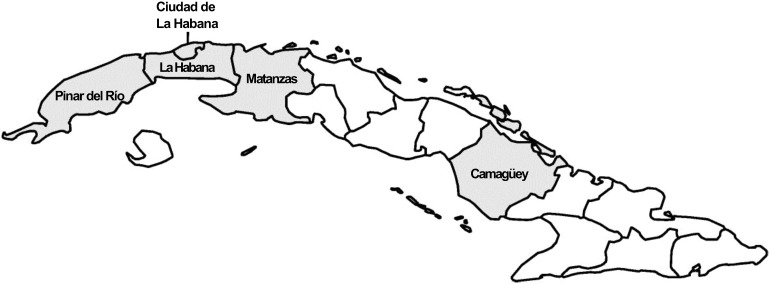
Map of Cuba showing the location of provinces surveyed (shaded) in the Cuba Prospective Study. Outline of provinces shown as they were at the time the baseline survey was conducted (1996–2002).

The study recruited 64 743 men and 81 813 women from five of Cuba’s 14 provinces (as they were at the time, before administrative boundary changes in 2011; Figure 1): Pinar del Río, La Habana, Ciudad de La Habana (Havana, the capital), Matanzas and Camagüey. The provinces were chosen to maximize the potential to investigate the associations of major risk factors with premature death. The selection criteria included: a wide geographical distribution in Cuba; a relatively settled population where it was reasonable to expect movement out of the area would be low; and a clear commitment and capacity from local staff. Collectively, the selected provinces accounted for just under half of Cuba's 11 million population in 2000. Within each province, publicly funded medical offices (which provide primary care for about 150 families in a defined geographical area^3^) were chosen randomly, using a computer-generated random allocation sequence, to participate in the study. Within each medical office, local staff (mostly the family doctor, but on occasion local nurses or other trained health care workers) visited households and invited all adults aged 30 and over to participate in the study. Overall, 74% of those registered with the selected medical offices (and eligible to participate) were recruited into the study.

## How often have they been followed up?

Participants are being followed up indefinitely for cause-specific mortality through Cuban death registries. In Cuba, all adult deaths are certified by a doctor, and the underlying and contributing causes of death are coded according to the International Classification of Diseases (using version 9 for deaths from 1996 to 2000, and version 10 for deaths from 2001 onwards). Mortality records for deaths throughout Cuba are obtained annually from the Ministry of Public Health database and are matched electronically to study participants using personal identifying information (national identification number, name, sex and date of birth). Partial matches (e.g. death records with the same identification number as a study participant but different name) were resolved by reviewing the original death certificate. Participants who have emigrated will be lost to follow-up, but due to Cuba’s migration laws, the proportion of participants who emigrate is estimated to be extremely low (<1% per annum).^4^ By 1 January 2017, 31 289 (21%) participants had died: 13 735 deaths (44% of all deaths) were due to cardiovascular diseases, 7329 (23%) to cancer, 8274 (26%) to other medical causes and 1842 (6%) to external causes; 109 (0.4%) were ill-defined. Only 241 (0.2%) participants are known to have emigrated.

## What has been measured?

For a large study such as this to be practicable and economically feasible in a resource-poor setting, the procedures need to be simple and streamlined. In this study, the full assessment at recruitment (including obtaining informed consent) took on average just 30–40 min to complete. After giving written consent, participants provided their national identification number and information on age, sex, education, occupation, lifestyle factors (including tobacco use and alcohol intake) and medical history (Box 1). Blood pressure was measured twice (once towards the beginning of the interview and once towards the end) with the participant seated, using a manual sphygmomanometer and standard techniques. Participants were invited to attend the medical office for measurement of height and weight, using a stadiometer and mechanical scales, respectively. Of those who had participated in the interview, 80% attended their local medical office for measurements. Interview data were entered manually into a computer in each province and sent to Oxford, where the datasets were merged. Identical copies of the combined dataset are held securely in Havana and Oxford. Between 2006 and 2008, 24 345 participants from medical centres in two areas (Colón and Jagüey Grande, both in the province of Matanzas) were re-surveyed using the same procedures as at recruitment. These repeat assessments will be used to account for random error and biological variation in measurements taken at baseline.^5^

**Box 1. Questionnaire data collected in the Cuba Prospective Study**

**Demographic data**

- Name
- Age
- Sex
- Ethnicity
- Marital status

**Socioeconomic data**

- Education
- Occupation

**Personal health behaviours**

- Smoking (cigarette, cigars)
- Alcohol (rum or other spirits; beer)

**General health-related data**

- Disease history (for 12 common conditions)
- Medications (for 15 common medicines)

**Measurements**

- Resting blood pressure (SBP/DBP measured twice)
- Height
- Weight

## What has it found? Key findings and publications

The majority of participants were recruited from two provinces: Matanzas (44%; Central Cuba) and Camagüey (40%; Eastern Cuba). Mean age at baseline was 55 (standard deviation [SD] 14) and 56% were women (Table 2). Most participants had received some formal education, but this was strongly related to age: almost all participants at younger ages were formally educated, whereas about one-fifth of those aged ≥70 reported no formal education.

**Table 2.** Baseline characteristics of 146 556 study participants of the Cuba Prospective Study, by age and sex

	Men, by age (years)				Women, by age (years)				All
	30−49	50−69	≥70	All	30−49	50−69	≥70	All	
Number of participants	27 232	26 600	10 911	64 743	35 537	33 028	13 248	81 813	146 556
Age, years (mean)	42.0	59.0	77.6	55.0	41.9	59.0	77.7	54.6	54.8
Region									
Matanzas (%)	44.4	44.7	44.3	44.5	43.6	43.0	42.6	43.2	43.8
Camagüey (%)	40.2	39.5	40.4	39.9	39.5	40.5	41.3	40.2	40.1
Pinar del Río (%)	8.7	8.4	6.1	8.1	9.0	7.3	5.5	7.7	7.9
Ciudad del La Habana (%)	4.7	5.4	6.9	5.4	5.9	7.1	8.7	6.8	6.2
La Habana (%)	2.0	2.1	2.4	2.1	2.0	2.1	2.0	2.1	2.1
Highest formal educational attendance
University (%)	15.0	7.1	2.1	9.6	14.1	4.8	2.0	8.4	8.9
High school/technical college (%)	44.1	29.1	10.9	32.3	38.2	18.9	6.4	25.2	28.4
Lower secondary (%)	27.9	28.3	16.2	26.1	30.7	28.0	14.0	26.9	26.5
Primary (%)	11.0	29.7	53.0	25.8	14.7	39.1	56.5	31.3	28.9
Less than primary (%)	2.0	5.8	17.7	6.2	2.3	9.1	20.8	8.0	7.2
History of one or more serious chronic diseases^a^									
Ischaemic heart disease (%)	1.8	6.1	10.4	5.0	1.8	6.9	10.7	5.3	5.2
Stroke (%)	0.5	1.9	6.1	2.0	0.6	2.1	6.0	2.1	2.0
Diabetes (%)	2.0	4.9	7.1	4.1	3.0	9.3	13.7	7.3	5.9
Cancer (%)	0.3	0.7	2.1	0.8	0.5	1.1	1.6	0.9	0.9
Other (%)	5.5	8.4	11.7	7.7	4.8	7.4	8.7	6.5	7.0
Any (%)	9.2	19.2	30.9	17.0	9.8	22.8	33.1	18.8	18.0

Missing values for highest educational attendance: men: 0.1%, women: 0.1%.

^a^Self-reported history of ischaemic heart disease, stroke, diabetes (type unspecified), cancer (any site), chronic obstructive pulmonary disease, chronic kidney disease, liver cirrhosis and peptic ulcer.

Overall, about one-fifth of participants reported a history of one or more specific chronic diseases. The prevalence of chronic disease was greater at older age and about one-third of those aged ≥70 reported one or more chronic diseases. The most prevalent conditions included ischaemic heart disease, stroke and diabetes (which was about twice as common in women than men). Reports of pre-existing cancer were rare in both men and women, and at all ages.

**Figure 2.**
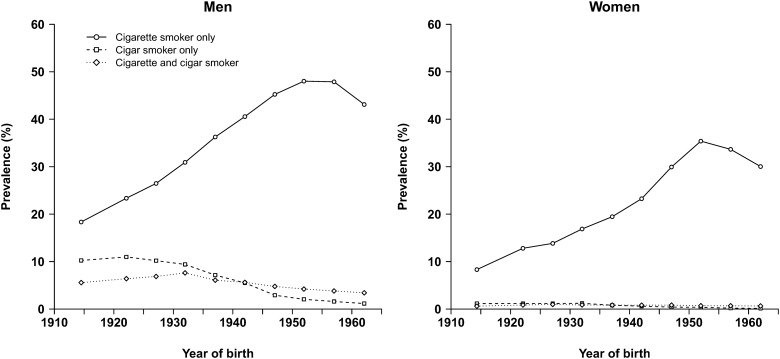
Prevalence of current smoking in the Cuba Prospective Study at baseline (circa year 2000), by sex, year of birth and type of smoking.

Table 3 reports the baseline distributions of selected factors by age and sex. About 60% of men and 30% of women reported ever smoking regularly (i.e. on most days for at least a year). The prevalence of ever smoking in men did not vary much by age, but was substantially higher in younger than older women. As in other low- and middle-income countries, stopping smoking was uncommon, with only 15% of ever smokers reporting quitting. As such, about one-half of men (49%) and one-quarter of women (26%) were current smokers at recruitment. Most current smokers reported smoking cigarettes exclusively (i.e. without cigars), with only 10% of men and <2% of women smoking cigars (exclusively or in combination with cigarettes). Cigar smoking was most common in older men, among whom 16% smoked cigars (i.e. about 40% of current smokers at that age) with or without cigarettes (Figure 2).

**Table 3.** Baseline distribution of selected risk factors for disease in 146 556 study participants of the Cuba Prospective Study, by age and sex

	Men, by age (years)				Women, by age (years)				All
	30−49	50−69	≥70	All	30−49	50−69	≥70	All
Number of participants	27 232	26 600	10 911	64 743	35 537	33 028	13 248	81 813	146 556
Current smoker (%)	52.0	50.2	38.7	49.0	33.8	23.5	12.8	26.3	36.3
Cigarettes only (%)	46.3	37.7	22.3	38.7	32.8	21.9	11.0	24.9	31.0
Cigarettes and cigars (%)	3.9	6.1	6.1	5.2	0.7	0.8	0.8	0.8	2.7
Cigars only (%)	1.7	6.5	10.3	5.1	0.3	0.8	1.1	0.6	2.6
Ex-smoker (%)	5.3	8.8	13.3	8.1	4.3	5.7	5.5	5.1	6.4
Never smoker (%)	41.7	39.1	45.3	41.3	61.1	70.0	80.7	67.8	56.1
At least weekly alcohol drinker (%)	35.8	26.4	11.7	27.9	6.3	3.4	1.6	4.4	14.8
Rum only (%)	18.2	15.7	7.3	15.3	2.1	1.6	0.7	1.7	7.7
Rum and beer (%)	15.4	9.4	3.8	11.0	3.1	1.4	0.6	2.0	6.0
Beer only (%)	2.2	1.3	0.6	1.6	1.2	0.5	0.2	0.7	1.1
BMI, kg/m^2^, mean (SD)	24.1 (3.6)	24.2 (3.9)	23.7 (3.9)	24.1 (3.8)	24.2 (4.2)	24.8 (4.6)	24.0 (4.6)	24.4 (4.5)	24.3 (4.2)
SBP, mmHg, mean (SD)	123 (12)	127 (15)	129 (17)	126 (14)	119 (14)	127 (17)	130 (18)	124 (16)	125 (16)
DBP, mmHg, mean (SD)	80 (8)	82 (10)	81 (10)	81 (9)	77 (10)	81 (11)	81 (11)	79 (10)	80 (10)

Missing values: smoking status (current or ex-smoker) of ever smokers: men: 1.6%, women: 0.8%; BMI: men: 1.0%, women: 2.7%; SBP/DBP: men: 0.1%, women: 0.2%.

A striking characteristic of this cohort is the proportion of smokers who started in childhood. Among current smokers, about one-third (men, 39%; women, 31%) reported smoking before the age of 15 years, and many before the age of 10. The effect on premature mortality of starting to smoke in early childhood has not been well characterized, and such analyses are planned for this cohort.

About one-quarter of men overall (and somewhat more at younger ages) reported drinking alcohol regularly (at least weekly), but prevalence of regular alcohol consumption was only about 5% overall in women. Most men drank rum only (55% of regular drinkers), or both rum and beer. Overall, mean systolic blood pressure, diastolic blood pressure and body mass index (SBP, DBP and BMI) were similar in men and women, and were much lower than in most Western countries at this time.^6^ Prospective analyses to quantify the associations of these major risks with cause-specific mortality in Cuba are ongoing.

## What are the main strengths and weaknesses?

This is the largest prospective study that has been undertaken in Cuba, and one of the largest studies in Latin America.^7^^,^^8^ The questionnaire was deliberately short (just one side of A4 paper, because of restrictions on the availability of paper at the time) so it was not possible to collect information on a wide range of risk factors. Questions were limited to several major risk factors only (i.e. smoking, drinking, adiposity and blood pressure) and information was not collected on the many other exposures of interest, such as diet and physical activity. As the study was designed to assess the risks associated with tobacco use, there was reasonably detailed information collected on smoking (including type of tobacco smoked, age started and age stopped, if relevant). Unfortunately, no information was collected on reasons for quitting, such as ill health, which have been shown to be valuable in other cohorts.^9^

Paperless data collection (including laptop- or internet-based methods) would have been more efficient and reliable than paper-based collection, but costs and technical capacity in Cuba at the time made such methods inappropriate. The study would also have benefited from collection and storage of biological specimens (including blood and urine) at baseline, but this was not possible owing to resource limitations.

The resurvey of over 20 000 participants is a key strength of this study, and will allow correction of the prospective associations for within-person variability over time of the major risk factors measured at baseline. This is particularly important in Cuba, where economic disruptions are likely to have affected the distribution of many of the risk factors measured at baseline. Indeed, the baseline survey was conducted towards the end of an economic crisis in Cuba, which began in 1991.^1^ The study also benefits from a well-developed public health surveillance system: causes of death are medically certified (meaning the number of deaths assigned to ill-defined causes is low) and the national identification system allows for efficient linkage of study participants to the national mortality database.

The Cuban health system was re-orientated in the 1980s towards primary care and preventive medicine. Despite limited resources, this system has had some notable achievements in public health, including low infant mortality, but mortality from the chronic diseases of middle age remains high. This cohort has now accrued sufficient events for reliable prospective analyses, and publication of the major findings on the associations of risk factors with cause-specific mortality are expected over the next few years. These findings will address uncertainties in the determinants of chronic disease in Cuba and will inform the ongoing efforts to prevent premature adult mortality in this population.

## Can I get hold of the data? Where can I find out more?

The study data are not freely available, but specific proposals for future collaboration are welcome, addressed to the study’s Cuba-based or Oxford-based investigators.

## Funding

This study was funded by core support from the UK Medical Research Council, British Heart Foundation and Cancer Research UK to the Clinical Trial Service Unit and Epidemiological Studies Unit and MRC Population Health Research Unit (both now part of the Nuffield Department of Population Health, University of Oxford). B.L. acknowledges support from the NIHR Oxford Biomedical Research Centre and the BHF Centre of Research Excellence, Oxford.
